# A Customized Mouthguard Design for a Child with Orofacial Myofunctional Disorder: A Case Report

**DOI:** 10.3390/pediatric18010016

**Published:** 2026-01-21

**Authors:** Masatoshi Otsugu, Fumikazu Tojo, Rena Okawa, Kazuhiko Nakano

**Affiliations:** Department of Pediatric Dentistry, Graduate School of Dentistry, The University of Osaka, Osaka 565-0871, Japan; tojo.fumikazu.dent@osaka-u.ac.jp (F.T.); okawa.rena.dent@osaka-u.ac.jp (R.O.); nakano.kazuhiko.dent@osaka-u.ac.jp (K.N.)

**Keywords:** mouthguard, children, dental trauma, orofacial myofunctional disorders, sports dentistry

## Abstract

When fabricating custom-made mouthguards for children, tooth replacement is an important factor for dentists to consider. In addition, orofacial myofunctional disorders and deleterious oral habits—such as incompetent lip seal and tongue thrusting—are relatively common among children and are associated with an increased risk of oral and dental trauma. Therefore, individual oral functional characteristics should be taken into account when designing custom-made mouthguards for pediatric patients. This report presents a case involving the design, fabrication, and appliance management of a custom-made mouthguard for a Japanese boy exhibiting incompetent lip seal and tongue thrusting. In this case, the anterior palate region of the mouthguard was left uncut, and multiple holes were created using a carbide bur to permit tongue–palate contact and provide sensory feedback related to tongue elevation. Over a 20-month follow-up period, no oral or dental trauma was observed. During appliance use, the patient demonstrated improved tongue elevation and an increased frequency of lip seal at rest. This case illustrates a custom mouthguard design that incorporates individual oral functional characteristics in a pediatric patient.

## 1. Introduction

A mouthguard (MG) is an appliance placed inside the mouth to reduce the risk and severity of oral injuries [1]. Wearing an MG is widely recommended, especially when engaging in contact sports [2]. For children and adolescents, custom-made MGs fabricated by a dentist are often appropriate because these patients’ mouths are still developing [2]. However, there is no clear standard regarding the fabrication or management of custom-made MGs in children, and very few reports exist—likely because of the difficulties involved in adapting a custom-made MG for tooth replacement [3,4,5]. We previously reported details of managing a child in the mixed dentition period with a custom-made MG and proposed a strategy for addressing tooth replacement [5].

Orofacial myofunctional disorder (OMD) refers to dysfunction of the lips, jaw, tongue, and/or oropharynx that interferes with normal growth, development, or function of other oral structures, leading to malocclusion and suboptimal facial development [6]. Among children, incompetent lip seal (ILS) is relatively common, with approximately 30% of Japanese children exhibiting it [7]. ILS and increased overjet are known risk factors for traumatic injury to the maxillary incisors [8]. Atypical swallowing, often associated with tongue thrusting, is another frequent OMD in children [9] and is linked to open bite, protrusion of the maxillary incisors, and increased overjet [10], which can further elevate the risk of dental trauma [8,11]. Therefore, when dentists fabricate and manage custom-made MGs for children, attention should be given not only to tooth replacement but also to individual oral functional characteristics.

The present case report describes the clinical use and appliance management of a custom-made MG in a Japanese child in the mixed dentition period with OMD. The objective of this case report was to illustrate the design and clinical application of a custom-made MG for a child with OMDs.

## 2. Case Report

**Figure 1:**
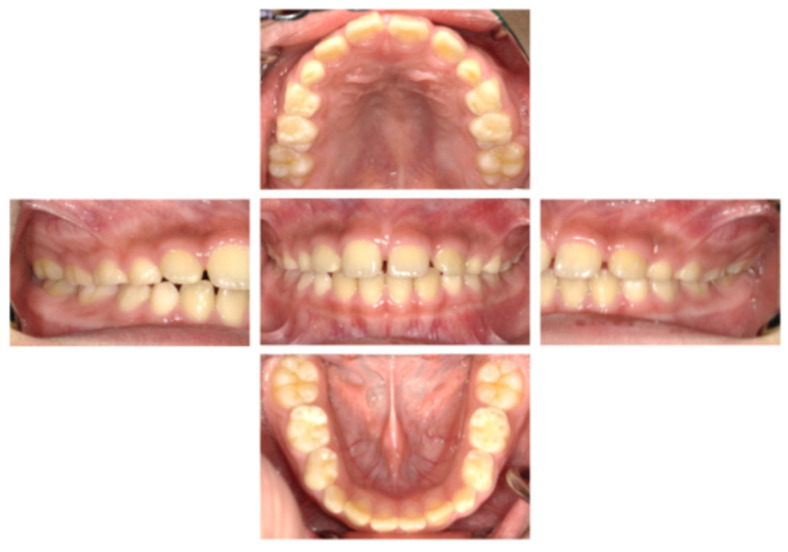
Intraoral examination findings (age 8 years 9 months).

**Figure 2:**
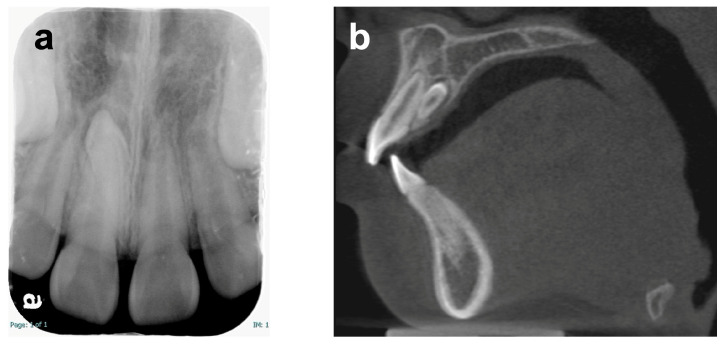
Dental radiography and cone-beam computed tomography imaging (age 8 years 9 months). (**a**) Dental radiograph; (**b**) Sagittal computed tomography image.

A Japanese boy aged 8 years 9 months was referred by a general dentist to the Pediatric Dentistry Clinic at the University of Osaka Dental Hospital for extraction of a supernumerary tooth. His medical, social, and family histories were unremarkable, and he had no prior experience with dental treatment. Intraoral and extraoral examinations revealed no obvious abnormalities such as dental caries, trauma, severe malocclusion, or soft tissue anomalies, including cleft lip and palate or ankyloglossia (Figure 1). Dental radiography and cone-beam computed tomography revealed a supernumerary tooth positioned on the palatal side of the maxillary right central incisor (Figure 2). At the age of 8 years 11 months, local anesthesia was administered, and the supernumerary tooth was extracted. One week later, the gingival healing prognosis was favorable.

**Figure 3:**
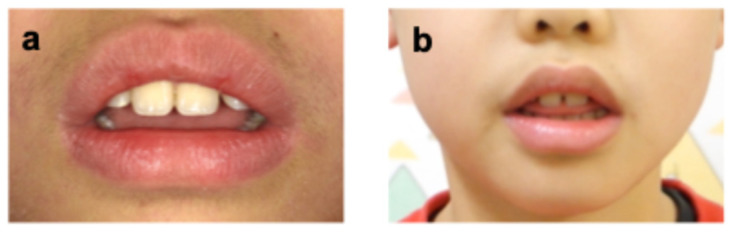
Extraoral photographs (age 9 years 0 months). (**a**) Incompetent lip seal at rest; (**b**) Pronouncing the/s/sound.

On the same day, the parent reported that the patient protruded his tongue when swallowing and speaking. Orofacial myofunctional evaluation revealed ILS at rest and tongue thrusting during swallowing and when pronouncing words containing/s/sounds (sigmatisms) (Figure 3). He was in the mixed dentition stage (Hellman’s dental developmental stage IIIA), with a Class I molar occlusal relationship, normal overbite (1.0 mm) and overjet (1.5 mm), and no crowding or caries, although the maxillary and mandibular incisors were slightly protruded. Additionally, he was unable to elevate his tongue voluntarily. Cone-beam computed tomography taken before extraction of the supernumerary tooth showed that his tongue was drooping in its resting position—a feature commonly observed in children with atypical swallowing (Figure 2b) [12]. He practiced two martial arts, Brazilian Jiu-Jitsu (mainly a ground-fighting system without protective gear) and Nippon Kempo (a direct-strike system using protective gear), each once a week. Although MGs are not mandatory for junior athletes in these sports, a custom-made MG was fabricated because of his high risk of oral and dental trauma, as well as the request of the patient and his parent.

**Figure 4:**
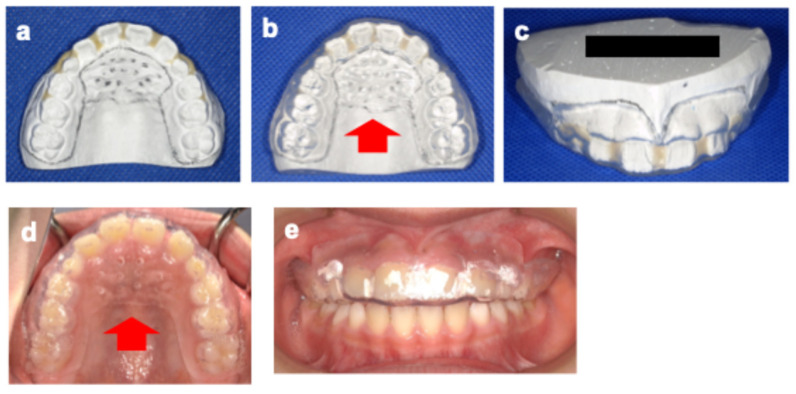
Fabrication of a custom-made mouthguard (MG) (age 9 years 1 month). (**a**) Occlusal view of the working model; (**b**) MG on the working model (occlusal view), arrowheads indicate multiple holes on the anterior palate; (**c**) MG on the working model (lateral view); (**d**) Worn MG (occlusal view), arrowheads indicate multiple holes on the anterior palate, arrowheads indicate multiple holes on the anterior palate; (**e**) Worn MG (lateral view).

Following the creation of a working model with dental stone (New Plastone^®^; GC Corporation, Tokyo, Japan), the interproximal space and labial surface of the model were filled with self-curing acrylic resin (Unifast II^®^; GC Corporation, Tokyo, Japan) to provide sufficient space for the eruption and movement of the maxillary permanent teeth (Figure 4a) [5]. A 3 mm-thick soft sheet (Bioplast^®^; Scheu-Dental, Iserlohn, Germany) was then heated and press-formed using a pressure forming machine (Erkopress 300Tp Plus^®^; Erkodent, Pfalzgrafenweiler, Germany). The outline of the MG was set 4 mm from the cervical margin on the buccal side to avoid interference with the frenum, and the posterior border was positioned at the distal aspect of the first molar. The sheet covering the anterior palate (mesial to the second deciduous molar and extending over the entire hard palatal fold) was left uncut, and multiple small holes (10 holes, 1 mm in diameter, spaced 6 mm apart) were drilled using a carbide bur (Sprint Carba HP SP2^®^; SHOFU, Kyoto, Japan) to permit tongue–palate contact and provide sensory feedback related to tongue elevation (Figure 4b–e). Occlusal adjustments were made to ensure bilateral contact in the primary and permanent molar regions. Follow-up examinations and orofacial myofunctional therapy, including tongue elevation exercises provided by the attending dentist, were scheduled every 2–3 months.

**Figure 5:**
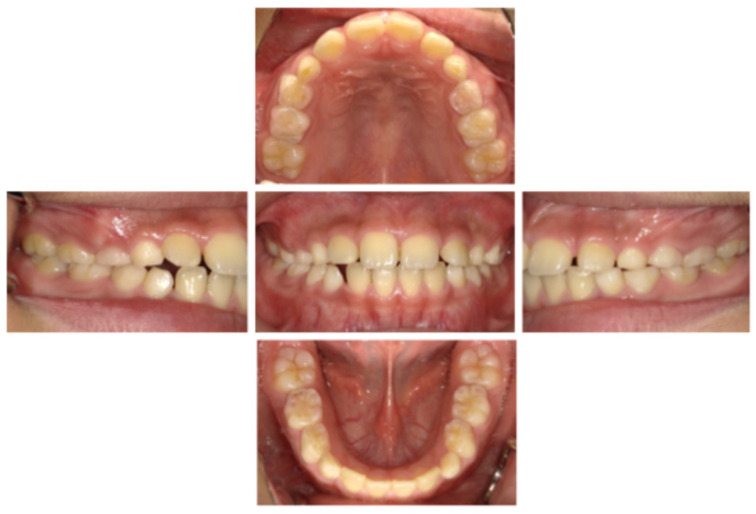
Intraoral photographs (age 9 years 11 months).

**Figure 6:**
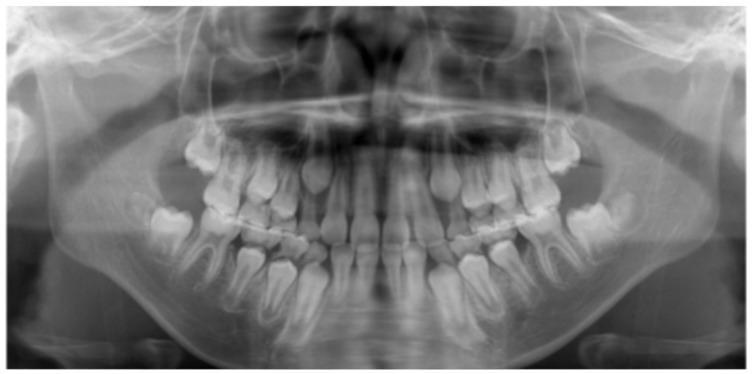
Panoramic radiography findings (age 9 years 11 months).

**Figure 7:**
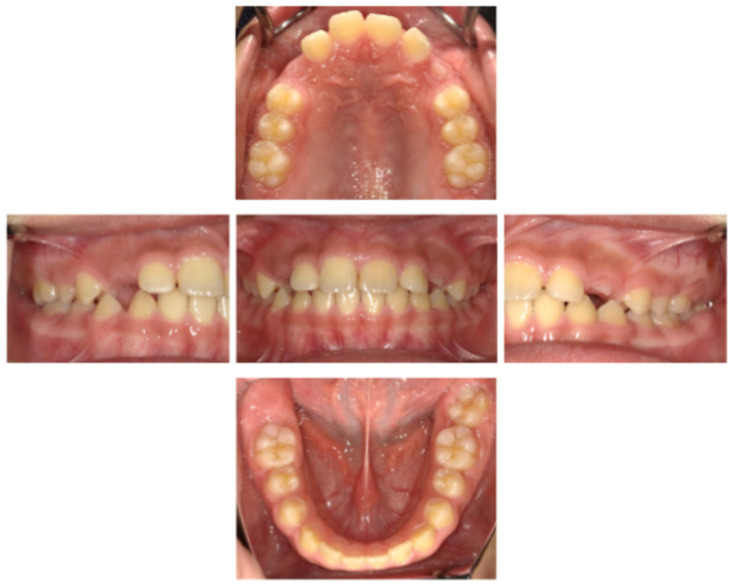
Intraoral photographs (age 10 years 9 months).

**Figure 8:**
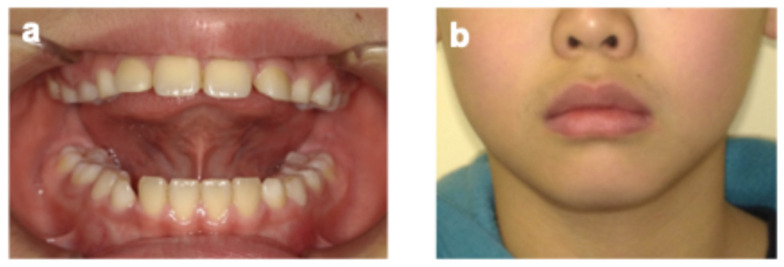
Orofacial myofunctional improvement (age 10 years 3 months). (**a**) Voluntary tongue elevation; (**b**) Improved frequency of lip seal at rest.

At the age of 9 years 11 months (10 months after MG fabrication), the patient-reported pain in the gingival area around the maxillary primary canine while wearing the MG. Intraoral examination and panoramic radiography revealed gingival swelling associated with the eruptive movement of the maxillary permanent canine (Figure 5 and Figure 6). Adjustment of the MG’s mucosal surface alleviated the discomfort. By the age of 10 years 9 months (20 months after MG fabrication), all primary teeth had exfoliated, and the patient had transitioned to permanent dentition (Figure 7). Throughout the course of management, he wore the MG for approximately 2 h, twice a week. No significant decrease in MG retention—assessed clinically based on the absence of dislodgement during mouth opening, speech, and light manual traction, as well as patient-reported stability—was observed. In addition, there was no obvious deterioration of the anterior palatal region over time, no hygiene-related complications, and no dental trauma during the 20-month follow-up period. During follow-up, he demonstrated improved tongue elevation, an increased frequency of lip seal at rest, and a lower frequency of tongue thrust during speech (Figure 8, Table 1).

Written and verbal informed consent for presentation of the case details was obtained from the patient’s parent.

## 3. Discussion

There have been no previous reports describing custom-made MGs for children that take into account individual characteristics such as ILS and tongue thrusting. In the present case, although the MG was fabricated according to our previous report [5], the sheet covering the anterior palate was intentionally left uncut, and multiple holes were drilled to provide sensory feedback related to tongue elevation. This modification was hypothesized to offer two potential benefits.

First, it may contribute to postural stability. Previous studies have suggested that impaired control of the center of gravity and reduced static balance during standing are risk factors for dental trauma [13], and that tongue elevation is associated with improved center-of-gravity sway and postural stability during isokinetic knee movement under experimental conditions [14]. One proposed mechanism is that tongue elevation may stimulate suprahyoid muscle activity, potentially influencing postural control through activation of the deep cervical flexor muscles [15]. Second, it may contribute to improved lip seal. ILS is a known risk factor for traumatic injury to the maxillary incisors [8]. Establishing appropriate tongue posture—specifically, contact of the tongue with the hard palate at rest—may support lip closure [16], and formation of a lingual–palatal seal may enhance orofacial proprioceptive input related to lip seal [17]. From this perspective, tongue elevation may contribute to improved lip competence. However, several important limitations regarding the two potential benefits must be acknowledged. In the present case, the mouthguard was used in conjunction with orofacial myofunctional therapy, and the individual contribution of the appliance cannot be distinguished from that of concurrent behavioral training. In addition, changes in tongue posture and lip seal were assessed clinically and based on observation rather than objective quantitative measures. Furthermore, although no dental trauma or falls were observed during follow-up, causal effects of tongue elevation or the mouthguard design on postural stability or trauma prevention cannot be inferred from a single case. Accordingly, these proposed mechanisms should be regarded as hypothetical and intended only to provide a conceptual framework for future investigation.

In the present case, this modification did not appear to have negative effects on safety or hygiene. Although the thickness and outline of the labial side of an MG affect its shock-absorbing capability [18], the present MG was not modified on the labial side. Conversely, its larger overall volume compared with standard MGs may help reduce the risk of accidental ingestion [19]. In addition, adequate hygiene was maintained through routine use of an MG cleaner and rinsing with water [20]. Nevertheless, careful monitoring of the oral mucosa remains important because the palatal coverage area of the MG is relatively large.

Managing a custom-made appliance during the mixed dentition period often presents challenges related to tooth replacement. In the present case, the patient experienced gingival pain while wearing the MG because of a gingival bulge associated with the eruption of the maxillary permanent canine, although the degree of eruption disturbance was minor, similar to our previous report [5]. The discomfort was temporarily relieved by adjusting the MG’s mucosal surface, and refabrication was ultimately unnecessary through the completion of tooth replacement. Because eruption disturbances of the maxillary canines are common in children [21,22], the timing of canine replacement is an important period during which adjustment or refabrication of custom-made MGs should be carefully considered.

Additionally, the presented MG design may be adaptable not only to custom-made MGs but also to boil-and-bite MGs. Although the FDI World Dental Federation encourages customization of MGs [23], boil-and-bite MGs are frequently used in children, particularly during the mixed dentition period, because of concerns related to tooth replacement. Regardless of MG type, dentists should play an active role in the selection, fitting, monitoring, and adjustment of MGs for pediatric patients.

In managing MGs for children, customized designs incorporating individual oral functional characteristics may be valuable for addressing the risk of oral and dental trauma.

## Figures and Tables

**Table 1:** Timeline of the clinical course.

Age	Clinical Events
8 years 9 months	First visit
8 years 11 months	Extraction of supernumerary tooth
9 years 0 months	Oral function examination and impression for MG fabrication
9 years 1 months	Initiation of MG use
9 years 11 months	Gingival pain associated with tooth eruption
10 years 9 months	Transition to permanent dentition

## Data Availability

The original contributions presented in this study are included in the article. Further inquiries can be directed to the corresponding author.

## Abbreviations

The following abbreviations are used in this manuscript:

MG	Mouthguard
OMD	Orofacial myofunctional disorder
ILS	Incompetent lip seal
